# Expression of P301L-hTau in mouse MEC induces hippocampus-dependent memory deficit

**DOI:** 10.1038/s41598-017-04305-4

**Published:** 2017-06-20

**Authors:** Xinghua Liu, Kuan Zeng, Mengzhu Li, Qun Wang, Rong Liu, Bin Zhang, Jian-Zhi Wang, Xiji Shu, Xiaochuan Wang

**Affiliations:** 1 Department of Pathophysiology, School of Basic Medicine and the Collaborative Innovation Center for Brain Science, Key Laboratory of Ministry of Education of China for Neurological Disorders, Tongji Medical College, Huazhong University of Science and Technology, Wuhan, China; 2 Department of Pathology and Pathophysiology, School of Medicine, Jianghan University, Wuhan, China; 3 Co-innovation Center of Neuroregeneration, Nantong, China; 4 Department of Neurology, Tongji Hospital, Tongji Medical College, Huazhong University of Science and Technology, Wuhan, 430030 PR China; 5 Department of Genetics and Genomic Sciences, Icahn School of Medicine at Mount Sinai, New York, NY 10029 USA

## Abstract

Intracellular accumulation of abnormally phosphorylated tau in different types of neurons is a pathological characteristic of Alzheimer’s disease (AD). While tau modification and associated neuronal loss and hypometabolism start in the entorhinal cortex (EC) in early AD patients, the mechanism by which mutant P301L hTau leads to dementia is not fully elucidated. Here, we studied the effects of P301L hTau transduction in the medial EC (MEC) of mice on tau phosphorylation and accumulation, and cognitive deficit. We found that the exogenous mutant tau protein was restricted in MEC without spreading to other brain regions at one month after transduction. Interestingly, expression of the mutant tau in MEC induces endogenous tau hyperphosphorylation and accumulation in hippocampus and cortex, and inhibits neuronal activity with attenuated PP-DG synapse plasticity, leading to hippocampus-dependent memory deficit with intact olfactory function. These findings suggest a novel neuropathological mechanism of early AD, which is initiated by tau accumulation in MEC, and demonstrate a tau pathological model of early stage AD.

## Introduction

The tauopathies, which characterized by hyperphosphorylation and aggregation of tau, are found in various neurodegenerative disorders such as frontotemporal dementia with parkinsonism linked to chromosome 17 (FTDP-17), Pick’s disease, and Alzheimer’s disease (AD)^1–3^. In patients with FTDP-17, site mutations of MAPT gene, cause prominent atrophy of the frontal and temporal lobes^4^, suggesting a crucial role of aberrant tau in neurodegeneration. In patients with AD, abnormal tau proteins are preferentially deposited in vulnerable brain regions, initially in entorhinal cortex (EC) and followed by the hippocampus, which is essential for learning and memory formation^5^.

It remains controversial whether expression of human P301L mutant Tau (P301L hTau) in EC could lead to amnesia. One study showed that expression of P301L hTau in EC of adult rats for 6 weeks induced memory deficit^6^, while another study demonstrated that the cognitive functions remained normal in transgenic EC-hTau mice, in which P301L hTau was overexpressed primarily in the EC^7^. The EC is considered as the original region with neuronal loss and hypometabolism in early AD patients^8, 9^. Metabolism and neuronal activities are disturbed in these patients. Positron emission tomography (PET) studies show that the regional cerebral blood flow (rCBF) in both asymptomatic AD and cognitively impaired patients significantly decreases in comparison with cognitively normal subjects^10^. Meanwhile, the perfusion changes measured with cerebral perfusion single photon emission tomography correlated with the Braak pathological stage in AD^11^. Using *in vivo* microdialysis, it has been shown that endogenous neuronal activity, represented by lactate concentration, can regulate the regional concentration of interstitial fluid Aβ and tau^12, 13^. However, whether and how expression of P301L hTau affects neuronal activity or induces toxicity has not been reported.

In the present study, we constructed recombinant adeno-associated viral vector GFP-P301L hTau (rAAV-GFP-P301L hTau) and the GFP vector without P301L hTau and injected them stereotaxically into the medial EC (MEC). After one month, we investigated the toxic effects of MEC expression of P301L hTau on hippocampus-dependent spatial memory and the neuronal activity. We found that expression of P301L hTau in MEC for a month induced hippocampal tau hyperphosphorylation and suppressed neuronal activity, resulting in impaired hippocampus-dependent memory with intact olfactory functions.

## Results

### Expression of P301L hTau in MEC induces endogenous hippocampal tau hyperphosphorylation and accumulation

**Figure 1:**
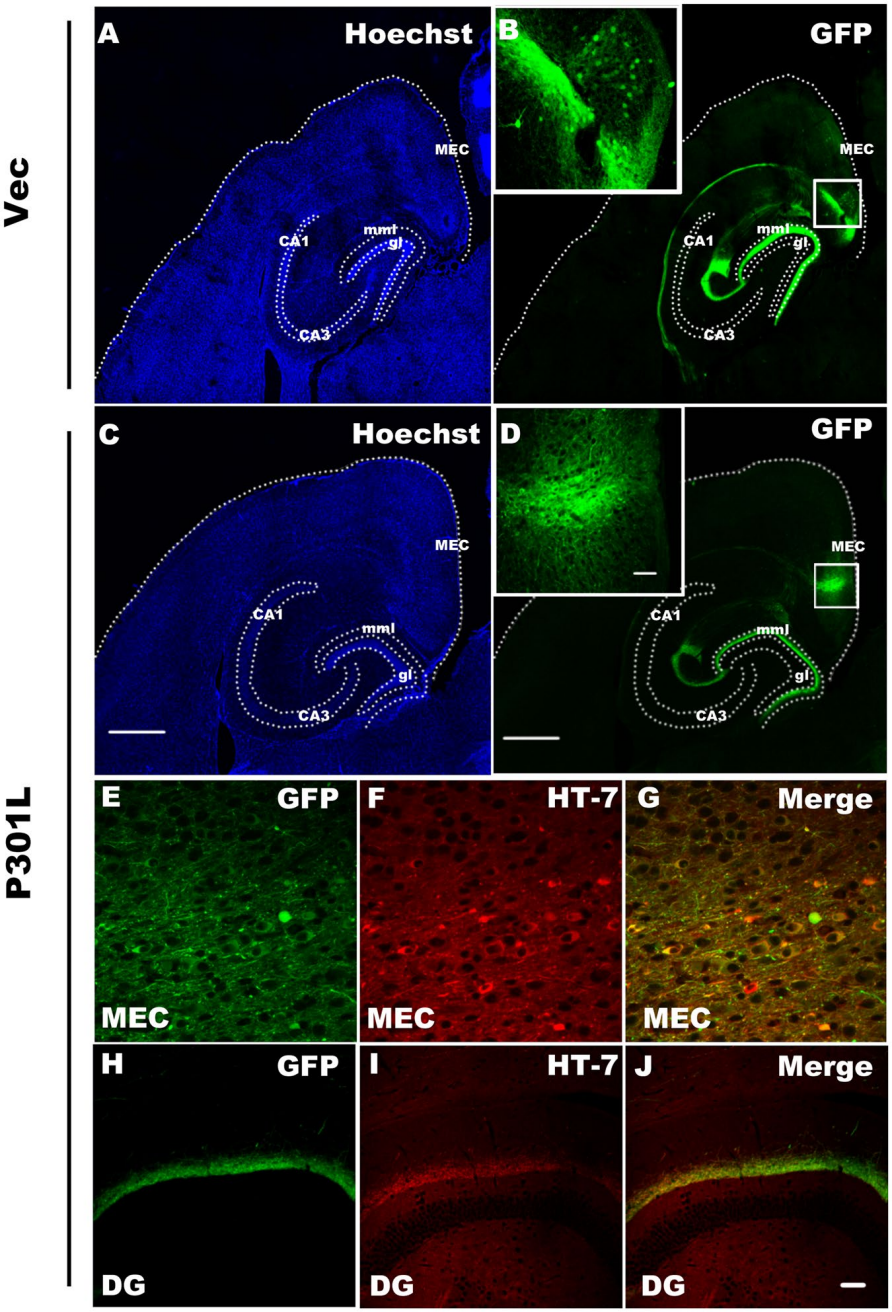
rAAV vehicles drives gene expression in the medial entorhinal cortex (MEC). Low-magnification view of a horizontal section of mouse brain stained with hoechst ((**A**) vector; (**C**) P301L) and GFP autofluorescence showing a restriction of target protein expression in MEC and its projection fibers to the middle molecular layer (mml) of hippocampal dentate gyrus (DG) ((**B**) vector; (**D**) P301L, arrows point cell bodies) one month after transduction via rAAV delivery, while no GFP was observed in the granular layer (gl) of the dentate gyrus, which is outlined with a white dotted line (scale bar, 500 μm). The insets in (**B**,**D**) show the high magnification of MEC (scale bar, 50 μm). HT-7 was employed to detect human tau in the present study. The results showed a co-localization of HT-7 with GFP in the neuronal cell bodies and fibers of MEC (**E**–**G**), and the projection fibers to DG region (**H**–**J**). (scale bar in (**A**–**D**) 500 μm; in insets of (**B**,**D**) 50 μm; in E-J, 50 μm).

The entorhinal cortex (EC) strongly and reciprocally connects with many other parts of the cerebral cortex such as hippocampal, temporal, and prefrontal cortex, making it a relevant node in the network mediating learning and memory^14^. The EC is invaded by abnormal tau at early stages of AD while the hippocampus is one of the first brain regions to suffer damage. Memory loss and disorientation are included among the early symptoms of AD^5^. To mimic an AD-like tau accumulation pattern, we constructed rAAV-GFP-P301L hTau and injected the viral vectors stereotaxically into the MEC subset of mice (3 m-old). Injection of the same volume of rAAV-GFP vector was used as control and the mice were sacrificed one month later to confirm the expression of tau protein. Immunofluorescent imaging showed robust green signals exclusively in the medial EC (MEC) region and its projection fibers to the middle molecular layer (mml) of hippocampal dentate gyrus (DG) in both vector (Fig. 1A,B and Fig. S1) and the P301L hTau expressing mice (Fig. 1C,D), with no staining in the granule cell layer (gl) of DG.

To confirm tau expression, we used HT-7, an antibody specifically recognizing human tau, and co-localization of HT-7 with GFP was detected in the neuronal cell bodies and fibers of MEC (Fig. 1E–G), and the projection fibers to DG region (Fig. 1H–J). No HT-7-positive signal was detected in the neuronal cell body of DG or any other subsets of the hippocampus (Fig. 1H–J), suggesting that the exogenous mutant tau protein was restricted in MEC without spreading to other brain regions.

**Figure 2:**
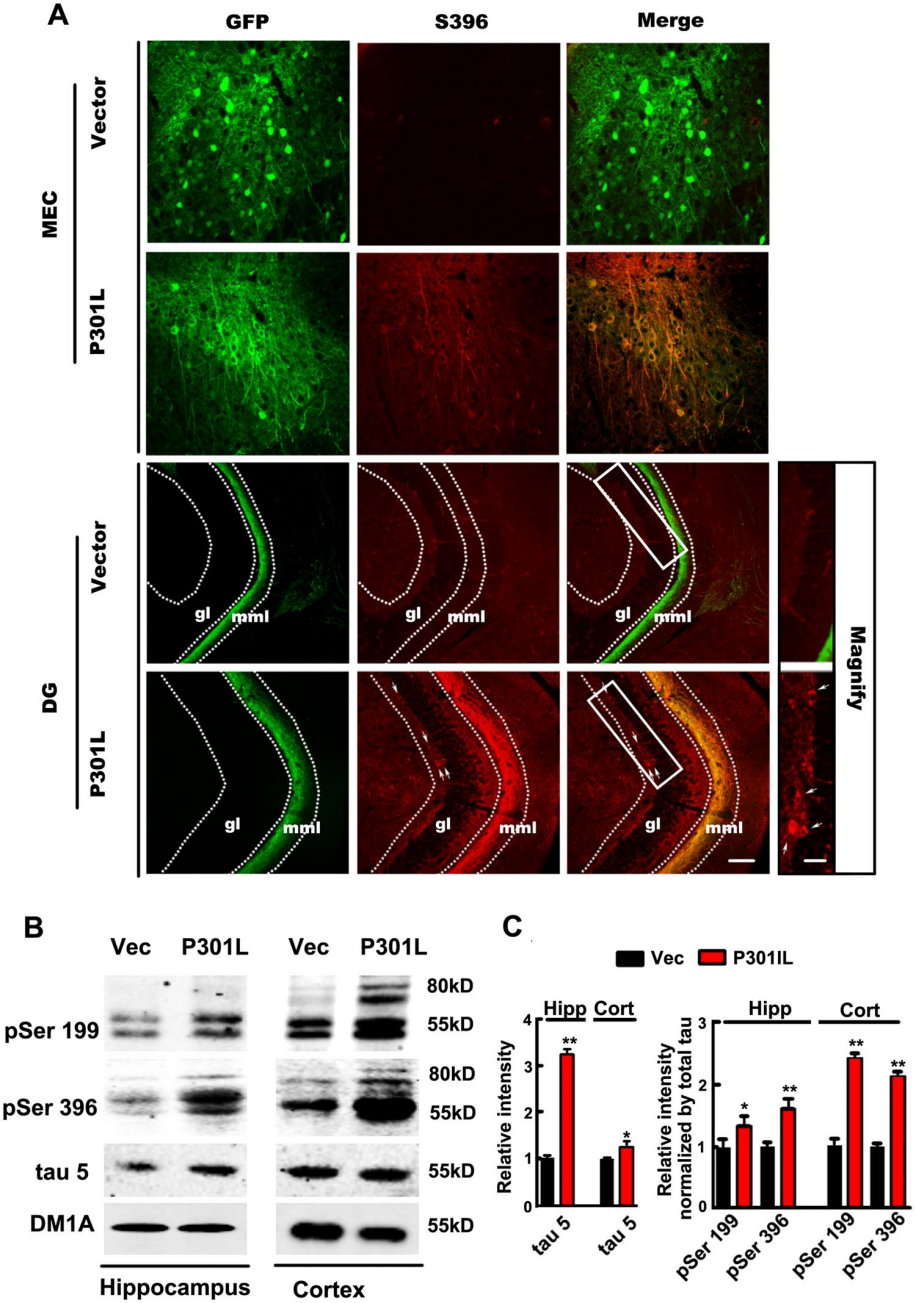
Expression of P301L hTau in MEC induces endogenous hippocampal tau hyperphosphorylation and accumulation. (**A**) Phosphorylation level of tau at Ser396 was significantly increased in MEC and its projection fibers to DG in brain slices one month after transduction of P301L hTau in MEC, where phosphorylated tau were co-localized with GFP. Interestingly, transduction of P301L hTau in MEC induced an increase of phosphorylated tau in the granular layer (gl) shown in magnified box, suggesting endogenous hippocampal tau hyperphosphorylation. (**B**) Western blotting showed that the phosphorylation of 55 kD tau at Ser396 or Ser199 was significantly increased in hippocampus and cortex, while only around 80 kD band (tau+GFP) was found in cortex of P301L mice. An increase of total tau probed by tau-5 suggested tau accumulation (n = 3 per group, *p < 0.05, **p < 0.01). Blots of Ser396 and Ser199 were overexposed here for the expressions of exogenous GFP labeled tau was much less than internal tau. Cropped blots are displayed here and full-length blots are included in the supplementary information. Data were expressed as mean ± SEM.

P301L hTau transgenic mice show tau hyperphosphorylation and accumulation in both cortex and hippocampus^15, 16^. To determine whether transduction of P301L hTau in MEC of mice induces tau hyperphosphorylation and accumulation, we employed phosphorylation site-specific tau antibodies, and found that the phosphorylation level of tau at Ser396 was significantly increased in MEC and its projection fibers to DG, whereas no significant hyperphosphorylation of tau was observed in other hippocampal regions (Fig. 2A). Interestingly, although exogenous mutant tau didn’t spread to the granule cell layer (gl) of DG (Fig. 1D), phosphorylated tau in gl was increased after transduction of P301L hTau in MEC (Fig. 2A lower right panels).

By Western blotting, increased phosphorylation of endogenous 55 kD tau at Ser396 or Ser199 was also detected in hippocampus and cortex, while only around 80 kD band of tau - GFP fusion protein was observed in cortex of P301L mice (Fig. 2B,C and Fig. S2A). To rule out the non-specific staining, we employed the secondary antibodies only as a negative control. We found that there were no staining with secondary antibody alone in western blot (Fig. S3A), and a very weak staining in immunohistochemistry (Fig. S3B–I). A western blot for GFP showed a 27 kD band in control mice while an around 80 kD band (Tau+GFP) was observed in P301L tau transduced mice (Fig. S2A). This suggests a good control for expression of the construct along the mice. Although there was no P301L hTau spread to the hippocampus and cortex, the total level of tau probed by tau-5 was also significantly increased compared with control, suggesting tau accumulation. Taken together, these findings imply that expression of the mutant tau in MEC induces endogenous hippocampal tau hyperphosphorylation and accumulation.

### Expression of P301L hTau in MEC impairs hippocampus-dependent memory with intact olfactory function

**Figure 3:**
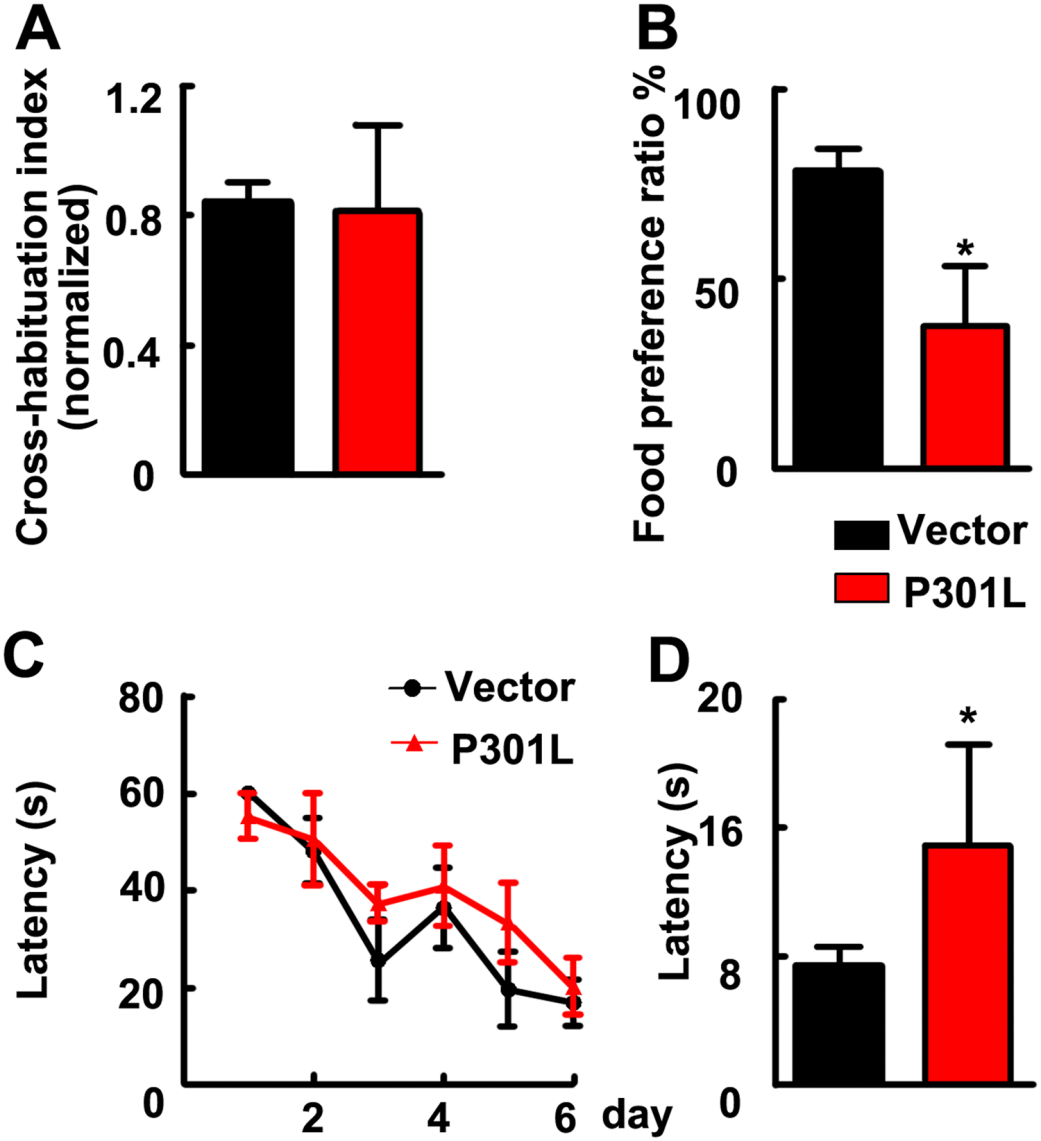
Transduction of P301L hTau in MEC for one month induced hippocampus-dependent memory deficits with an intact olfactory function. (**A**) In odor cross-habituation test, the olfactory function was intact in both groups revealed by cross-habituation index. (**B**) The STFP test showed that the olfactory-dependent memory was attenuate significantly in P301L hTau transduced mice compared to control. (**C**) In the MWM test, escape latency of training indicated that learning capacity of P301L hTau transduced mice kept normal like control mice. (**D**) The probe test showed that the expression of P301L hTau in MEC impaired hippocampal-dependent spatial memory of mice. (vector, n = 8, P301L, n = 11. *p < 0.05). Data were expressed as mean ± SD.

As EC is bidirectionally connected with olfactory and hippocampus, we investigated whether expression of tau affects olfactory- and/or hippocampus-dependent behaviors. Firstly, we performed odor cross-habituation test, and found no significant difference in cross-habituation index between the control and P301L hTau-expressing groups (Fig. 3A), suggesting that mutant tau transduction in MEC does not influence the olfactory function of the mice. Next, STFP test detecting hippocampus-dependent non-spatial olfactory memory was carried out. We found that the ratio of eating powdered chow scented with cinnamon to total food consumption was decreased remarkably in the mice expressing P301L hTau (Fig. 3B), suggesting impairment of the hippocampus-dependent non-spatial olfactory memory. Finally, we used MWM test to measure the effects of P301L hTau on hippocampus-dependent spatial learning and memory. No difference in finding the hidden platform was detected between P301L hTau and the control groups during 6 days learning trials (Fig. 3C), however, the latency to locate the target quadrate in P301L hTau-transduced-mice was significantly increased in the probe trials after removal of the platform compared to control group (Fig. 3D), which confirms the impairment of spatial memory retrieval, as a possible result of tau hyperphosphorylation and accumulation. These data together demonstrate that expression of P301L hTau in MEC for only one month can mimic AD-like hippocampus-dependent memory deficits.

### Expression of P301L hTau in MEC suppresses neuronal activity without causing significant cell death

**Figure 4:**
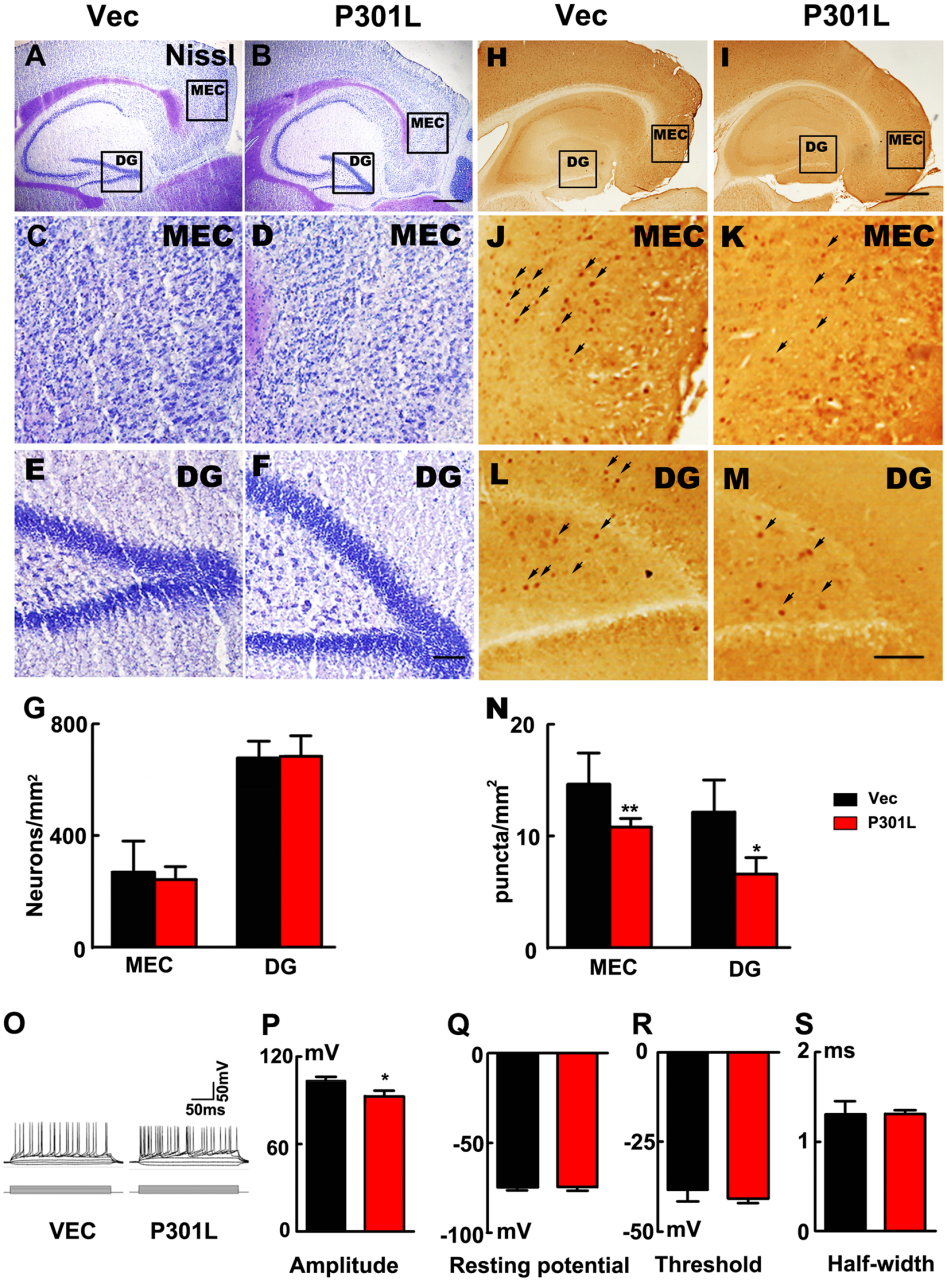
Expression of P301L hTau in MEC suppresses the neuronal activity without causing significant cell death. (**A**–**F**) Nissl staining indicated that the numbers of neurons were not changed in P301L hTau transduced mice in both MEC and DG regions compared to control (scale bar in A and B, 500 μm; in C-F, 100 μm). (**G**) Quantitative analysis of the Nissl staining showed that P301L hTau transduction didn’t lead to neuronal death (n = 3 per group). (**H**–**M**) c-fos was employed to detect the neuronal activity. A density of the c-fos puncta in both MEC and DG subsets was remarkably decreased in P301L hTau-transduced mice compared with the control. (scale bar in H and I, 500 μm; in J-M, 100 μm). (**N**) Quantification of the number of c-fos positive cells per 1 mm^2^ revealed that P301L hTau lowered the c-fos expression in the MEC and DG. (n = 3 per group, 9 slices, *p < 0.05, **p < 0.01). (**O**–**S**) Whole-cell patch results suggested that AP amplitude was significantly lowered in P301L hTau cells compared to control (**O**,**P**). However, passive properties were not altered in P301L hTau cells since their membrane resting potential (**Q**) was unchanged compared to controls. There was no difference in threshold (**R**) or half width (duration, (**S**)) between control and P301L hTau cells (n = 3 per group, 9 slices, *p < 0.05). Arrows in J-M were used to indicate the cFos staining. Data were expressed as mean ± SEM.

Previous studies showed neuronal loss in 5.5-month-old and older rTg4510 mice and P301L hTau transgenic mice^17^. In the present study, we did not observe significant neuronal loss in the MEC and the DG as shown by Nissl staining after one-month expression of tau (Fig. 4A–G). These data indicate that short-term expression of P301L hTau may not cause a general cell death.

**Figure 5:**
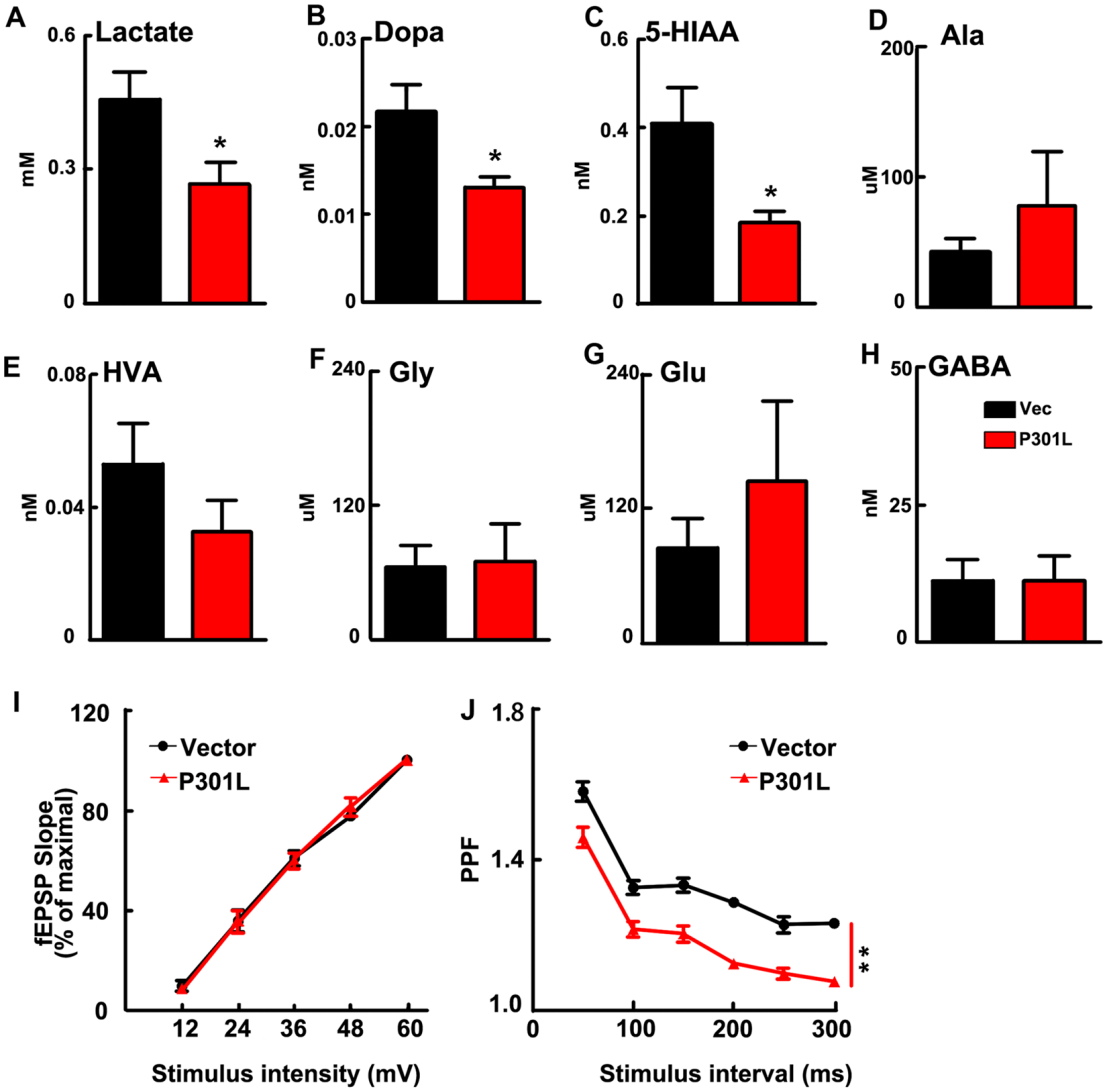
P301L hTau transduction in MEC decreases the probability of presynaptic transmitter release. (**A**) Microdialysis results indicated the lactate level was significantly reduced. (**B**,**C**) The levels of dopamine and 5-HIAA (monoamine neurotransmitter or the metabolites) were also decreased in the intercellular space, while the levels of Ala, Hva, Gly, Glu and GABA were not changed (**D**–**H**). (n = 6 per group, *p < 0.05). (**I**) Input-output curve of the EC-DG perforant path fibers. (**J**) Mutant P301L hTau produced a decrease of PPF in PP-DG pathway (n = 3 per group, 9 slices, **p < 0.01). Data were expressed as mean ± SEM.

The characteristic features of abnormal tau-associated neurodegeneration are chronic and progressive^16–18^, implying that tau may suppress neuronal activity. To test this hypothesis, we measured the expression level of the regional brain c-fos, an immediate early gene sensitive to the alteration of neuronal activity^19^. We found that density of the c-fos puncta in both MEC and DG subsets was remarkably decreased in mice expressing P301L hTau compared with the control (Fig. 4H–N), suggesting an inhibition of MEC and DG neuronal activity. We also used whole-cell patch recording to measure the regional neuronal activity in brain slices. We observed that the action potential (AP) amplitude was significantly decreased in P301L hTau neurons compared to the control (Fig. 4O,P), while the resting membrane potential, threshold and half width (duration) were unchanged compared to the controls (Fig. 4Q–S). Since the extracellular level of lactate represents neural activity^12^, we then used *in situ* microdialysis and measured the lactate level in MEC subset of the mice with P301L hTau transduction or the control mice. We found that the lactate level was significantly reduced (Fig. 5A), simultaneously, the levels of dopamine and 5-HIAA (metabolite of 5-HT) were also decreased in the intercellular space (Fig. 5B,C), while the levels of Ala, HVA, Gly, Glu and GABA were not changed (Fig. 5D–H).

To explain the decrease of dopamine and 5-HIAA, paired pulse facilitation (PPF) were measured in the perforant path fibers (PP)-DG synapses. PPF is a form of short-term plasticity traditionally used as an indirect way to estimate the probability of transmitter release (Pr). Synapses with low Pr usually show an impaired PPF^20^. In the present study, electrophysiological recordings showed that expression of P301L hTau in MEC did not modify the input/output ratio of the EC-DG perforant path fibers (Fig. 5I). However, Mutant P301L hTau lowered PPF significantly (Fig. 5J), which suggested a decrease in the probability of presynaptic transmitter release in MEC such as dopamine and 5-HIAA.

These data together strongly suggest that expressing P301L hTau in MEC decreases the neuronal activity in both MEC and the hippocampus.

### Expression of P301L hTau in MEC suppresses synaptic plasticity of PP-DG

**Figure 6:**
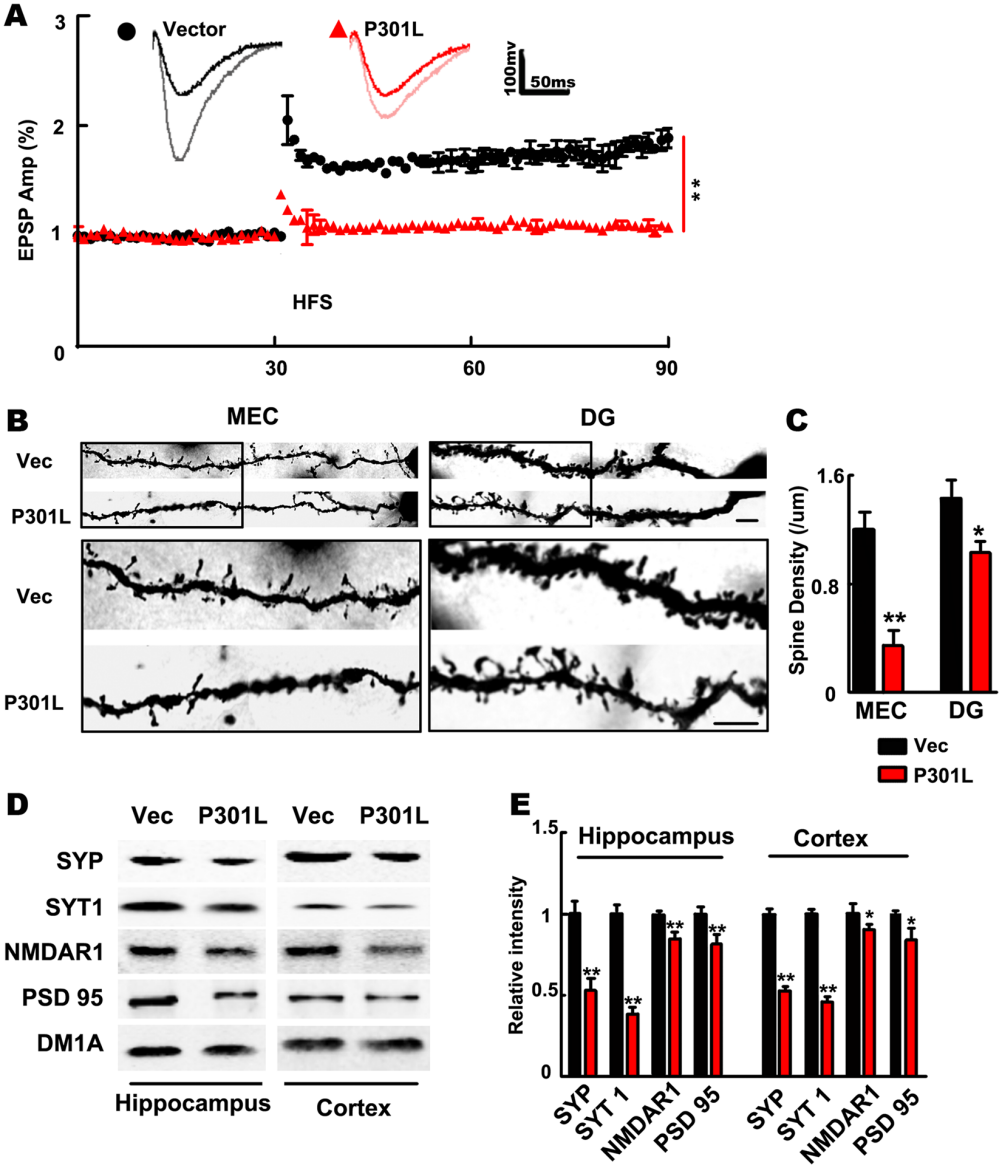
Expression of P301L hTau in MEC inhibits synaptic plasticity of PP-DG. (**A**) The Long-term potentiation of PP-DG was inhibited in P301L hTau transduced mice. The inset graphs represent for each group an example of unit fEPSP before (dark line) and after (light line) LTP induction, which revealed that LTP induction is reduced in P301L hTau transduced mice (HFS, high frequency stimulation. n = 3 per group, 9 slices **p < 0.01). (**B**) Golgi-stain revealed that the MEC spine density was decreased in MEC and DG of P301L hTau transduced mice. (**C**) Quantitative analysis of spine density. (n = 3 per group, 9 slices, **p < 0.01. Scale bar, 10 μm). (**D**,**E**) Levels of synaptophysin and synaptotagmin I (presynaptic proteins) and NMDAR1 and PSD95 (postsynaptic proteins) were decreased in both the hippocampus and cortex of P301L hTau transduced mice (n = 3 per group, *p < 0.05). Cropped blots are displayed here and full-length blots are included in the Supplementary Information. Data were expressed as mean ± SEM.

Based on our observation of no alteration of the input/output ratio of PP-DG and impaired PPF, we further studied whether expression of P301L hTau affects synaptic plasticity. By electrophysiological recording, we observed that expression of P301L hTau in MEC diminished high frequency stimulation-induced LTP compared with the control mice (Fig. 6A), suggesting that expression of the mutant tau impairs synaptic plasticity and suppresses the evoked presynaptic neurotransmitter release (Fig. 5B,C,J). Golgi staining showed an apparent decreased spine density in both MEC and DG of the P301L hTau-transduced mice (Fig. 6B,C). These data together suggest that expression of the mutant tau impairs both pre- and post-synaptic plasticity.

To gain further insight into the molecular mechanism underlying the plasticity impairment, we analyzed the synapse-associated proteins by Western blotting in hippocampus and cortex. We found that levels of synaptophysin and synaptotagmin I (presynaptic proteins) and NMDAR1 and PSD95 (postsynaptic proteins) were decreased in both cortex and the hippocampus (Fig. 6D–E and Fig. S2B).

## Discussion

Increasing evidences suggest that tau proteins play a crucial role in neurodegeneration. For instance, in an autosomal dominantly inherited dementia termed FTDP-17, the site mutations of tau gene (such as P301L or P301S) alone cause neurodegeneration and behavior abnormalities^15, 21^. Expression of mutated P301L tau in the hippocampus progressively impaired performance on a hippocampal-dependent spatial memory task^22^. In fact, tau pathologies initially appear at entorhinal cortex in AD patients, which has direct neural projections to hippocampus^5^. However, it is unclear whether expression of human P301L mutant Tau in EC is associated with dementia. In the current study, we observed cognitive deficits one month after transduction of P301L hTau in mouse MEC. EC is bidirectionally connected with olfactory bulb and hippocampus. Its early dysfunction might impair olfactory- and hippocampus-dependent behaviors^23^. Therefore, we test olfactory function and cognition by odor cross-habituation test, STFP and MWM. The odor memory is acquired in a single session and can last for weeks after training in rodents^24–26^. Lots of evidences demonstrated that the hippocampus is one of the brain regions that are implicated in the STFP. Hippocampal c-fos is necessary for long-term memory of a STFP^27–29^. NMDA receptors, muscarinic receptors and acetylcholine are crucial for the persistence of socially acquired, hippocampus-dependent, non-spatial memory^30–32^. Meanwhile, orbitofrontal cortex and medial prefrontal cortex also contribute to STFP^29, 33^. In our study, the mice expressing mutant P301L hTau had intact olfactory function as demonstrated by odor-habituation test, while STFP showed lower food preference. The results of MWM and STFP indicated that expression of P301L hTau in MEC for a month could cause impaired hippocampus function.

Injection of AAV-P301L tau in the hippocampus of wild-type FVB/N mice mediated neurodegenerative process without formation of large fibrillar tau-aggregates or tangles, but with increased expression of cell-cycle markers^34^. Intracerebroventricular injection with AAV1-P301L tau led to the accumulation and deposition of hyperphosphorylated and abnormally folded tau species in NFTs and neuritic inclusions^35^. Microinjection of AAV-P301L tau into mouse lateral EC has been described as a model of early-stage Alzheimer disease^36^. These mice developed tau hyperphosphorylation, neuronal degeneration, and synapse loss within a few weeks, in contrast to the transgenic mice with spatially restricted human tau expression, which require 18 months or longer to develop perforant pathway damage^37^. We analyzed the phosphorylation status and distribution of tau by biochemical and immunohistochemical methods in the present study. These results suggested that expression of the mutant tau in MEC induced endogenous tau hyperphosphorylation and accumulation in hippocampus and cortex.

The expression of immediate early gene c-Fos, which is involved in mechanisms associated with the maintenance of memory^38^, was decreased not only in MEC, but also in DG. These results indicated that short-term abnormal tau expression not only affects local c-fos levels, but also c-fos in projective regions as well. Decreased c-fos expression might partially explain why/how P301L hTau expression in MEC induced hippocampus-dependent behavioral impairments. In the present study, the decreased neuronal activity due to pathologic tau was indicated by reduced levels of early gene c-fos, ISF lactate, neurotransmitters and electrical activities. Normal neuronal function is sustained by utilizing lactate aerobically as its sole energy substrate^39–41^. Neural activity triggers release of the neurotransmitter glutamate that is taken up into the astrocyte, and stimulates the breakdown of glycogen, the uptake of glucose, and glycolysis, to produce lactate^42^. Here, we analyzed dialysate of MEC and found that P301L hTau led to significant decline of lactate levels. The findings indicated that the MEC neuronal activities were impaired after transduction of abnormal tau. Using microdialysis, we found that expression of P301L hTau in MEC led to a decline in levels of dopamine and 5-HIAA which is a metabolic product of the inhibitory neurotransmitter 5-HT. This is consistent with an impaired PPF, which suggested a decrease in the probability of presynaptic transmitter release in MEC.

Electrophysiological recordings of the EC-DG perforant path fibers showed normal input-output curves in EC neurons, suggesting that there were no drastic effects of P301L hTau on basal synaptic transmission. The perforant pathway projection from the EC to the DG is critically important for long-term memory. Meanwhile, this projection also develops tau and amyloid pathologies and progressive degeneration starting in the early stages of AD^5, 36^. Our data showed that mutant P301L hTau resulted in an impairment of PP-DG LTP, which might be the cause of the dysfunction of memory retrieval.

Mutant P301L hTau decreased action potential amplitude but did not change the membrane resting potential, excitability threshold and action potential half width. These results are inconsistent with Crimins JL’ works^43^. They found significant electrophysiological changes including a depolarized resting membrane potential, and increased action potential firing rates-all indicative of hyperexcitability, and these changes presented in the early stage and sustained in the advanced stage in both atrophic and intact neurons in the rTg4510 mouse model of progressive tauopathy. These discrepancies might be due to short-term expression (only one month) and local transduction in MEC. In an APP and tau P301L transgenic mouse model, glutamate was increase in the hippocampal synaptic cleft and synaptic dysfunction in CA1 region was impaired^44, 45^. In the present study, an increasing trend in glutamate level was found in the intercellular space of P301L transduced mice, but no significant difference when compared with the control animals. We found that mutant P301L hTau induced a decrease of both MEC and DG spine density suggesting impaired synaptic plasticity in aberrant tau affected MEC region.

In summary, aberrant tau induced a reduction of local neural activity and impaired PP-DG synapse plasticity, which might be the neuropathological mechanism of early stages of AD. We here provided a model of early tau pathological stage of Alzheimer’s disease by delivering mutant P301L hTau to MEC and found cognitive dysfunction.

## Materials and Methods

### Ethics statement

All methods were carried out in accordance with the approved guidelines. All experimental protocols were approved by the Tongji university institutional committee. The study was reviewed and approved by the China national institutional animal care and use committee.

### Subjects

Three-month old male C57BL/6 mice (Experimental Animal Central of Tongji Medical College) were housed with *ad libitum* access to food and water. The mice were kept in the facility at 25 °C with 60% relative humidity under a 12-h light/dark cycle.

### Recombinant adeno-associated virus (rAAV) production

cDNA encoding full-length 441–amino acid human 4 repeat tau bearing a P301L mutation with fusion protein GFP or GFP expression plasmids were cloned into a rAAV9 vector, which had been described previously^45^. rAAV vectors expressing P301L hTau or GFP under the control of the CAG promoter, a woodchuck post-transcriptional regulatory element and the bovine growth hormone polyA were generated by plasmid transfection with AAV helper plasmids in HEK293T cells. Forty-eight hours after transduction, the cells were harvested and lysed in the presence of 0.5% sodium deoxycholate and 50 U/ml Benzonase (Sigma, St. Louis, MO) by freeze thawing, and the viruses were isolated using a discontinuous iodixanol gradient. The genomic titer of each virus was determined by quantitative PCR.

### Stereotaxic injections

For stereotaxic injections, the mice were deeply anesthetized with chloral hydrate by intraperitoneal injection (30 mg/kg) and positioned in a stereotaxic apparatus. Then, 1 μl of viral vectors (10^11^ viral genomes/ml in PBS) were injected bilaterally into the MEC (Bregma −4.8 mm, lateral ±2.8 mm, depth 3.5 mm) at a flow rate of 0.1 μl/min by a micro syringe connected to a monitored nanoinjector. The pipette remained in place at the injection site for an additional 5 minutes before slow removal. After surgery, the mice were injected penicillin (200,000 U, i.m.) to prevent infection.

### Morris water maze (MWM) test

Spatial learning and memory were measured by MWM test. The temperature of the room and the water kept at approximately 22 °C. The maze consisted of a circular pool (1.2 m in diameter) filled with titanium dioxide dyed water. A platform (10 cm in diameter) was submerged 1 cm below the surface of the water in the third quadrant. For spatial learning, mice were trained in water maze to find a hidden platform for 6 consecutive days, 4 trials per day with a 30-min interval from 2 pm to 8 pm. On each trial, the mouse started from one of the middle of the four quadrants facing the wall of the pool and ended when the animal climbed on the platform. The mice were allowed to search for the platform for a maximum of 60 s, after which they were gently guided to the platform if they did not find the platform. The mice were allowed to remain on the platform for 30 s. The swimming pathways and escape latencies of the mice to find the hidden platform were recorded by a video camera fixed on the ceiling. On the seventh day, the memory of mice was assessed by a 60-s probe test where the platform was removed from the pool.

### Social transmitted of food preference (STFP) test

Hippocampus-dependent non-spatial olfactory memory was studied using the STFP task. The experiment was conducted in three phases: (i) habituation to flavored food, (ii) interaction between ‘demonstrator’ and ‘observer’ mice, and (iii) test of the food preference in the ‘observer’ mice. In the habituation phase, untreated mice as the demonstrators were housed singly in a cage for 24 h with free access to water but not food. At the end of 24 h, each demonstrator was allowed to eat powdered chow scented with either cinnamon (1%, w/w) or cocoa (2%, w/w) for 1 h. The criterion for inclusion in the experiment was consumption no less than 0.2 g. Each demonstrator was then placed with observer mice, control and P301L hTau transduced mice for 30 minutes. After the interaction period, the demonstrator mouse was removed from the interaction cage and returned to its individual cage. Observer mice were food deprived but water *ad libitum* individually in the same laboratory. In the final phase of the experiment, the food preference of the observer mice was tested 24 h after the end of the interaction with the demonstrator. The one-hour preference test consisted of presenting each observer with a pair of weighed flavored food in the individual cage. Both food powder were removed and weighed to quantify the food preference of the observer mice at the end of the final phase. The ratio of the weight of the cued food eaten was used as a measure of food preference. The measure of food preference was calculated by: [Demonstrated Food Consumed (g)/Total Food Consumed (g)] × 100].

### Odor cross-habituation test

Mice were screened for olfactory deficits using an odor-cross habituation test according to previous study^46^. Odors (n = 6, limonene, isoamyl acetate, ethyl acetate, vanillin, chamomile, rose essential oil) were diluted 10^−3^ in mineral oil and applied to a cosmetic tips. Each odor was delivered by inserting the odor stick into a port on the top of the animal’s home cage for 4 successive trials, 60 s each, separated by 60 s inter-trial intervals. The duration of time spent in investigating, defined as snout-oriented sniffing within 1 cm of the odor presentation port, was recorded across all trials by a single observer blind to genotypes.

### *In vivo* microdialysis

Animals were subjected to a stereotaxic surgery to insert a cannula into MEC (AP −4.8 mm, ML 2.9 mm, and DV −2.5 mm relative to bregma under anesthesia). Cannula was implanted in the brain vertically through a small drilled aperture in the skull and fixed with dental cement. 24 h after the surgery, a CMA7 microdialysis probe (1-mm membrane length, 6-kDa cut off, mean recovery 32 ± 5.6%) was inserted into the cannula. The probe was connected to a syringe pump and perfused with artificial cerebrospinal fluid (ACSF, containing in mM: 124 NaCl, 2.5 KCl, 2 CaCl_2_, 2 MgSO_4_, 1 NaH_2_PO_4_, 25 NaHCO_3_, and 10 Glucose; pH 7.4) at a flow rate of 0.8 μl/min for 60-min equilibration period and then the dialysis fractions was collected per 30-min for at least 3 h into Eppendorf tubes containing 2 μl of antioxidant solution (0.1 M perchloric acid). The perfusates were either collected in 0.1 M perchloric acid (contained 0.4 mM sodium metabisulfite) for immediate quantification of dopamine, homovanilic acid (HVA), and 5-HIAA content with HPLC, or stored at −80 °C until lactate glycine (Gly), glutamate (Glu), alanine (Ala) and GABA were measured. After each experiment, the animal was anaesthetized and sacrificed. The brains were fixed with paraformaldehyde, and horizontal sections were sliced and stained with toluidine blue to ensure the proper location of the guide cannula in right EC. Animals with incorrectly placed cannula were excluded from the analyses.

### High Performance Liquid Chromatograph (HPLC)

Dopamine, 5-HIAA and HVA were analyzed with HPLC-Electrochemical Detector (HPLC-ECD). The samples were filtered by 0.2 μm glycosylated hair acid sample processor before injection. The standards of dopamine, 5-HIAA and Hva (HPLC grade, Sigma Company) were dissolved in the solution with 0.01% L-cysteine, 0.01 M HClO_4_, 0.5 mM Na_2_EDTA. All separations were performed on a MD2150 column (150 mm × 3.2 mm, 2.0 μm) and a Shiseido C18 pre-column (10 mm × 3 mm, 3 μm) at 30 °C. The DA, 5-HIAA and HVA were eluted in sequence in 20 min and detected by an electrochemical detector. Ala, Gly, Glu and GABA were analyzed by HPLC-Fluorescence Detector (HPLC-FLD). Trazodone was added in the samples to a concentration of 29.2 μg/ml and homogenized as internal standard substance. Before injection, 250 μl samples were reacted with 100 μl NaHCO_3_ (2 M) and 200 μl dansyl chloride acetone solution (10 g/L) in 80 °C water bath for 30 min, and then stopped by 100 μl acetic acid acetone solution (1 M). The standards of Ala, Gly, Glu and GABA (HPLC grade, Sigma) were dissolved in 0.2 M NaHCO_3_. All separations were performed on a Kromasil C18 (4.6 × 250 mm, 5 μm) at room temperature. The mobile phase was composed of 42% methanol, 1% tetrahydrofuran, 14 mM sodium heptanesulfonate, and the pH was adjusted to 4.2 with glacial acetic acid. The flow rate was 1 ml/min. The Amino acid reaction products were eluted in sequence and detected by fluorescence detector at 500 nm when activated by 360 nm laser light.

### Lactate assay

An enzymatic lactate assay kit (BioVision) was utilized to measure lactate present in microdialysis samples according to the manufacturer’s instructions. Assays were read on a Bio-Tek Synergy 2 plate reader at 570 nm.

### Electrophysiology

The brain of anesthetized mouse was rapidly removed and transferred to ice cold, oxygenated (95% O_2_, 5% CO_2_) ACSF. Brain was horizontal sliced with a pre-chilled vibratome to produce 350 µm sections. The slices were incubated in oxygenated ACSF at 29 °C for at least 90 min to recover. After recovery, a single slice was transferred to a MED probe (MED-P515A, 8 × 8 array, interpolar distance 150 μm, Panasonic) and positioned in such a way that the perforant path fibers (PP, stimulating region) and DG region (recording region) covered most of electrodes, immersed with 29 °C oxygenated ACSF, stabilized carefully with a meshed anchor, and allowed to equilibrate for at least 30 min. Oxygenated fresh ACSF was continuously perfused at the rate of 2 ml/min with the aid of a peristaltic pump during the entire experiment. Field excitatory postsynaptic potentials (fEPSPs) were recorded at perforant path fibers-DG (PP-DG) synapses. Electrical stimulation was delivered to one channel located within the PP, and evoked fEPSPs were monitored and recorded from the other 63 channels. The stimulation intensity was approximately 40% of intensity that induced the maximal fEPSPs. For long-term potentiation (LTP) recording, baseline responses were evoked for at least 30 min until stabilized, followed by a high frequency stimulation (HFS) protocol (consisted of 10 bursts, each containing 4 pulses at 100 Hz with an inter-burst interval of 200 ms) was given at a stimulation intensity that was adjusted to elicit 40% of the maximal response. After HFS, the test stimulus was repeatedly delivered once every 1 min for at least 1.5 hour to record LTP. In another set of experiments, paired-pulse facilitation (PPF) was recorded prior to HFS. The ratio of the slope of the second response to the slope of the first response was calculated and averaged. The interpulse interval varied between 50, 100, 150, 200, 250 and 300 ms.

For whole-cell patch, all data were acquired by using a Multiclamp 700B amplifier, digitized with a Digidata 1440 and analyzed by pClamp 10.0 (Molecular Devices, Sunnyvale, CA, USA). Data were filtered at 5 kHz with a low-pass Bessel filter and digitized at between 5 and 20 kHz. Conventional visually-guided whole-cell patch recordings were obtained from MEC neurons and a pipette with a resistance of 3–5 MΩ. Recordings were conducted in a submerged recording chamber perfused (2 ml/min) with the ACSF at room temperature. All neurons included in this study had a resting membrane potential below −55 mV and series resistance were tested after completion of each recording, and recordings were not analyzed for neurons with access resistance larger than 25 MΩ or if >20% changes was detected following the recording. To analyze the action potential properties, neurons were recorded at resting or at −70 mV membrane potentials and depolarized with 200-ms current steps in 20-pA increments.

### Golgi-cox staining

Brains were placed directly into Golgi solution (1 g potassium chromate, 1 g mercuric chloride, 0.8 g potassium chloride and 100 ml double-distilled water) where they remained in a bottle kept in dark for 6 weeks. Thereafter, the solution was exchanged sequentially in 10, 20 and 30% sucrose solutions in light-protected jars to aid in maintaining histological structure. The brains were sectioned at 100 μm thickness with a vibratome and placed onto gelatin-coated glass slides. After rinsing with double-distilled water, slides were incubated in ammonium hydroxide for 30 min. Following a water wash, the slides were incubated for 30 min in a black and white film developer diluted 1:9 with water and then rinsed with final double-distilled water. Slides were mounted with resin and cover-slipped. All subsequent quantitative analyses were conducted in an unbiased manner to group designation with repeated measurements.

### Immunohistochemistry and immunofluorescence

Horizontal sections of 30 μm thickness were permeabilized for 30 min by a mixture of 0.5% Triton X-100 in PBS and 0.3% H_2_O_2_, and blocked with 3% BSA/PBS for 1 hour at room temperature. Sections were incubated with c-fos (1:200, cell signaling) primary antibody diluted in blocking solution overnight at 4 °C. Antibody-antigen complexes of c-fos were visualized using horseradish peroxidase-conjugated secondary antibody and the DAB chromogen system. After immunohistological staining and washing, slices were mounted on microscopic slides and dried naturally, followed by dehydrated, transparentized and sealed with coverslips. For immunofluorescence, brain slices were incubated with primary antibodies (PS396, Signalway Antibody; HT-7, Innogenetics. Dilution, 1:200) overnight at 4 °C. Slices were washed with PBS and incubated with Alexa Fluor 568-conjugated or cy3 secondary antibodies in PBS containing 1% BSA for 1 h. Cell nuclei were visualized with Hoechst 33258 (2.5 μg/ml final concentration). Slices were mounted onto glass slides and sealed using 50% glycerin in PBS. Immunohistochemistry images were acquired with an Olympus photomicroscope using 4× and 10× objectives. Immunofluorescence images were scanned using a Zeiss LSM 710 laser scanning confocal microscope.

### Western blot

Hippocampal and cortical protein extracts were separated on 12% SDS polyacrylamide gels and transferred to nitrocellulose membranes at 276 mA for 1 h. The membranes were blocked with 3% BSA in PBS, and incubated overnight with primary antibodies at 4 °C. Antibodies used in our study were DM1A (T9026, sigma), synaptophysin (MAB368, Millipore), Synaptotagmin I (ab13259, abcam), PSD95 (#2507, cell signaling), NMDAR1 (AB9864, Millipore), GluR1 (04-855, Millipore), PS396 (11102, Signalway Antibody), PS199 (AB9652, Millipore), GFP (ab1218, abcam) and Tau-5 (MAB361, Millipore). The membranes were incubated with fluorescent secondary antibodies in 3% BSA for 1 h at room temperature and washed. Odyssey system was used for color development and analysis of blots. In particular, the band used for the quantifications of PS199 and PS396 includes all bands among 55–80 KD.

### Statistical analysis

All data were analyzed using the GraphPad Prism version 5.04 statistical package. For odor cross-habituation, the raw investigatory values were normalized to the maximum investigatory duration per animal for each odor (max during trials 1–4). The maximum investigation duration was assigned a value of ‘1’ and the lesser investigation times a proportion of 1. In order to calculate cross-habituation index, the normalized investigatory values from all 4th trial odor presentations were subtracted from the following 1st trial odor presentations. Mann-Whitney non-parametric test was used and statistical significance was accepted at the level of P < 0.05.

## Electronic supplementary material

supplementary information
