# Supplementary material for: Expression of P301L-hTau in mouse MEC induces hippocampus-dependent memory deficit
Source: Sci Rep. 2017 Jun 20;7:3914. doi: 10.1038/s41598-017-04305-4 (PMC5478664; doi:10.1038/s41598-017-04305-4)
Supplement: Supplementary file 1 — supplementary information [file 41598_2017_4305_MOESM1_ESM.pdf]

Expression of P301L-hTau in mouse MEC induces hippocampus-dependent memory deficit

Xinghua Liu [a,d, 1], Kuan Zeng [a,1], Mengzhu Li [a], Qun Wang [a], Liu Rong [a], Bin Zhang [e], Jian-Zhi Wang [a,c], Xiji Shu[* b], and Xiaochuan Wang[* a,c]

[a]Department of Pathophysiology, School of Basic Medicine and the Collaborative Innovation Center for Brain Science, Key Laboratory of Ministry of Education of China for Neurological Disorders, Tongji Medical College, Huazhong University of Science and Technology, Wuhan, China

[b]Department of Pathology and Pathophysiology, School of Medicine, Jianghan University, Wuhan, China.

[c]Co-innovation Center of Neuroregeneration, Nantong, China

[d]Department of Neurology, Tongji Hospital, Tongji Medical College, Huazhong University of Science and Technology, Wuhan 430030, PR China

[e]Department of Genetics and Genomic Sciences, Icahn School of Medicine at Mount Sinai, New York, NY 10029, USA

[1]These authors contributed equally to this work

[*]Correspondence to:

Dr. Xiji Shu, Department of Pathology & Pathophysiology, School of Medicine, Jianghan University, Wuhan 430056, P. R. China. Tel.: 86-27-84230413; Fax: 86-27-84225149; E-mail: shuxiji@sina.com

Dr. Xiaochuan Wang, Department of Pathophysiology, Tongji Medical College, Huazhong University of Science and Technology, Wuhan 430030, P. R. China. Tel.: 86-27-83692625; Fax: 86-27-83693883; E-mail: wxch@mails.tjmu.edu.cn

Supplementary Information

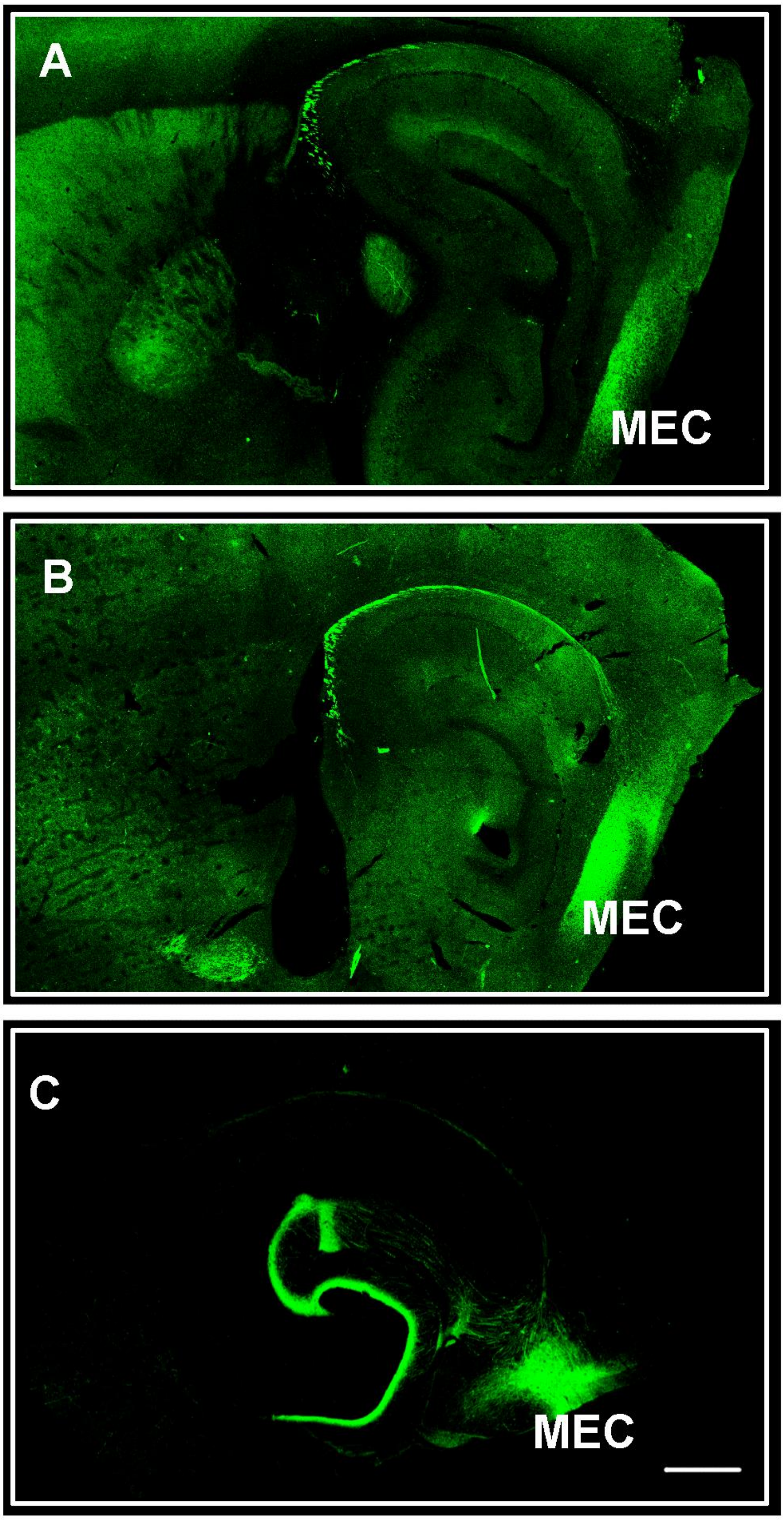

Supplementary Figure S1. (A, B) Representative views of brain sagittal sections and (C) horizontal section. GFP autofluorescence showing a restriction of target protein expression in MEC. Scale bar=500 μm.

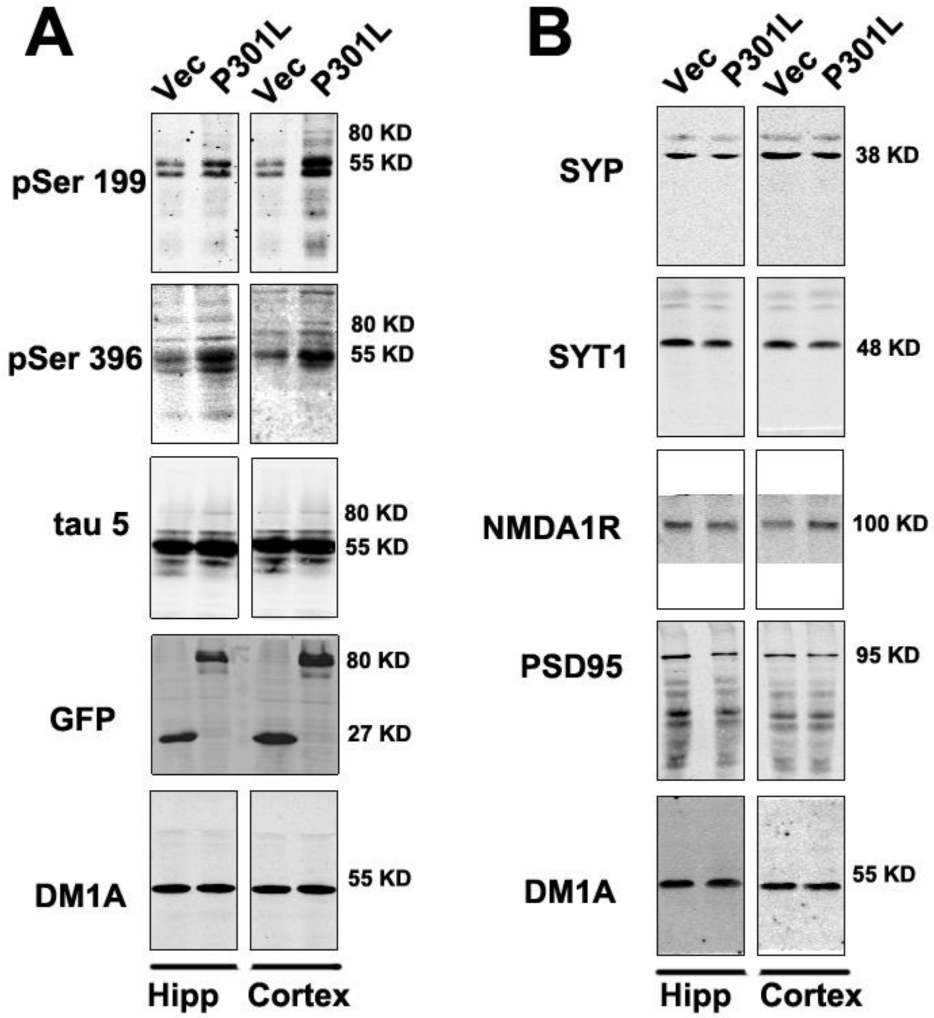

Supplementary Figure S2. (A) Full-length blots and mutiple exposures of figure 2B. Blot for GFP. (B) Full-length blots of figure 6D.

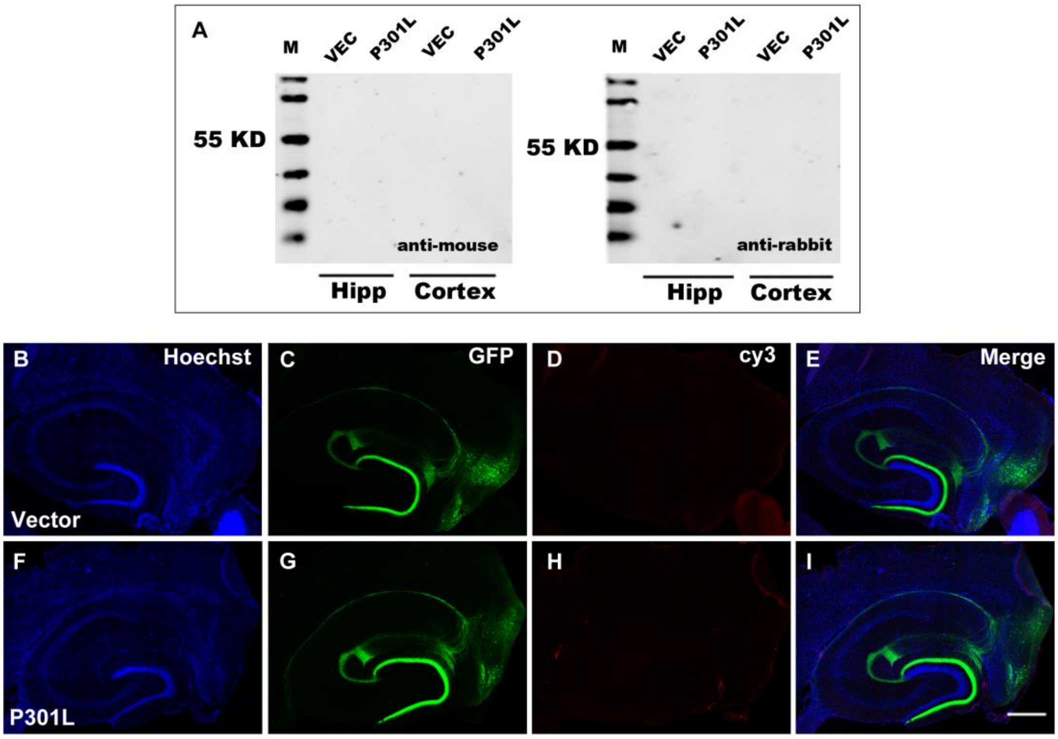

Supplementary Figure S3. (A-H) Horizontal brain sections of vector and P301L mice stained with secondary antibody (cy3) only as a negative control (scale bar, 500 μm). (I) Western blot with anti-mouse or anti-rabbit secondary antibody as a negative control (M: protein marker).
